# Stroke in Young Adults: A Prospective Study from Northwestern Nigeria

**DOI:** 10.5402/2012/468706

**Published:** 2012-02-16

**Authors:** L. F. Owolabi, A. Ibrahim

**Affiliations:** Neurology Unit, Department of Medicine, Aminu Kano Teaching Hospital, Bayero University, PMB 3452, Kano 700, Nigeria

## Abstract

*Background*. Stroke is an important cause of morbidity and mortality in young adults especially in developing countries. This two-centre prospective study aimed at reviewing the pattern, types, and case fatality of stroke in the young adults in Northwestern part of Nigeria. *Methods*. Consecutive patients aged 18–40 years admitted to the medical wards of two tertiary hospitals from June 2008 to August 2010 were recruited. Relevant clinical data were obtained from the patients.The survivors were followed up in neurology clinics for 6 months. *Results*. A total of 71 patients aged 18–40 yrs, (mean age was 31.9 ± 6) comprising 52(73%) males and 19(23%) females were enrolled. Forty two (59.1%) patients had infarctive stroke. The risk factors included hypertension (74.7%) patients, smoking (50.7%), hypercholesterolemia (9.9%), non-hypertensive cardiac diseases (8.5%), HIV (8.5%), diabetes mellitus (4.2%) cocaine and amphetamine (2.8%), and sickle cell disease (2.8%). Only twelve (17%) patients presented within the first 6 hours of onset of stroke. Seventeen (23.9 %) patients died, case fatality in the first 24 and 72 hrs was 4.2% and 19.7%, respectively. *Conclusion*. Our data suggests that stroke in young adults is not as uncommon as previously suggested. Hypertension, smoking, hypercholesterolemia, cardiac diseases and HIV are the most common risk factors.

## 1. Introduction

Stroke is a major cause of morbidity and mortality worldwide [1]. Although stroke is predominantly a disease of the middle age and the elderly, its occurrence in younger age groups is not rare. Stroke is an important cause of morbidity and mortality in young adults, especially in developing countries [2]. Stroke in the young is particularly tragic because of the potential to create a long-term burden on the victims, their families, and the community at large. Stroke in adults under the age of 45 results in a greater loss of potential years of life than for older adults [3].

In spite of the huge socioeconomic impact of stroke in this age group, there is a scarcity of data regarding stroke in young adults in the northwestern Nigeria. Most of the data available originated from southwestern Nigeria; they were obtained over two decades ago and were retrospective. Moreover, stroke in adult below 45 yrs of age was reported to be uncommon [4, 5].

It was against this background that we embarked on this multicentre prospective study aimed at reviewing the pattern, types, and case fatality of stroke in the young adults in the northwestern part of Nigeria.

## 2. Methods

In this prospective study, consecutive patients aged 18–40 years who were admitted to the medical wards of the two tertiary hospitals; Aminu Kano Teaching Hospital (AKTH), Kano, and Murtala Muhammad Specialist Hospital, (MMSH) Kano, from June 2008 to August 2010 were recruited in the study. Eligibility for the study was in accordance with the World Health Organization (WHO) definition of stroke as ‘rapidly developing clinical signs of focal or global disturbance of cerebral function, with symptoms lasting 24 hours or longer or leading to death, with no apparent cause other than vascular origin' (WHO 1989) [6]. All the patients enrolled were assessed by clinicians experienced in the subtlety of stroke diagnosis, typing, and management. Laboratory investigations like random blood sugar (RBS), full blood count (FBC), retroviral screening (RVS), electrolyte, urea, creatinine, and so forth were done. Classification of stroke into haemorhagic and infarctive stroke was based on computerized tomography of the brain (CT Brain) or magnetic resonance imaging (MRI). The World Health Organization criteria with diagnostic accuracy of 71% were also used in about half of the patients [7].

A questionnaire was designed to extract relevant clinical data from the patients. The questionnaire recorded the age, sex, date of admission, delay before presentation, reasons for delay, time of death, accompanying symptoms, stroke type, and the predisposing factors. Only those who had complete information and met the World Health Organization criteria for the clinical diagnosis of stroke were included. The survivors were followed up in neurology clinics for 6 months, status of disability on admission and at discharge was recorded using modified Rankin disability scale [8].

Management of the patients in these centres was in accordance with Aminu Kano Teaching Hospital guideline on stroke management, which is a modification of American Heart Association/American stroke association (AHA/ASA) guidelines [9] and more recently Nigerian stroke guidelines [10].

The case fatality at 24 hours and 72 hours was recorded. Analysis of data was done using the statistical software package SPSS version16. Descriptive statistics were depicted using absolute numbers, simple percentages, range, and measures of central tendency (mean, median) as appropriate. The Chi-square test was used to test the significance of associations between categorical groups. Statistical significance was fixed at probability level of 0.05 or less.

## 3. Result

A total of seventy-one stroke patients aged 18–40 yrs, comprising fifty-two (73%) males and 19 (23%) females, were enrolled; mean age was  31.9 ± 6  yrs; twelve (17%) patients presented at the hospitals within the first 6 hrs of developing a stroke. Twenty-nine (40.8%) patients had CT brain, while four (5.6%) had magnetic resonance imaging (MRI). Forty-two (59.2%) were ischaemic and twenty-nine (40.8%) were haemorhagic. There was transient ischaemic attack (TIA) prior to stroke in 29% and the commonest TIA type was motor (53.3%).  Table 1 shows distribution of stroke type across sex and age group. Only twelve (17%) of the patients presented to accident and emergency unit within the first 6 hours of onset of stroke (Table 2), out of which only three (4.2%) were Infarctive stroke.The commonest risk factor identified was hypertension as it was present in fifty three (74.7%) of the patient, this was followed by smoking found in thirty-six (50.7%) patients (Table 3) out of whom twenty (55.6%) were current smokers and sixteen (44.4%) were former smokers. Seventeen (23.9%) patient died; case fatality was 4.2% and 19.7% in the first 24 and 72 hrs, respectively. Out of those that died aspiration pneumonitis was documented in 22% of them as associated cause of death. Median hospital stay was seventeen days. Forty-eight (62%) of the survivors were seen on follow up 6 months after the onset of stroke, no cases of death at home were reported (Table 4).

## 4. Discussion

Stroke is not uncommon among young people, as was previously assumed in early reports [3, 5]. Our experience from the two tertiary hospitals in Kano, northwestern Nigeria, further substantiates this premise. Our result showed that 29.3% of 242 cases of stroke seen during a 2-year and 8- month period were aged 40 years or less. This finding is in conformity with the report of Nwosu et al. in a study at the University of Nigeria Teaching Hospital in Enugu, in southeast Nigeria, which found incidence of 27.9% among hospital inpatients [4]. However, most of our cases were in the 31- to 40-year-age range; this finding still corroborates the general observation that the incidence increases with age and age being the most powerful independent predictor of cardiovascular morbidity and mortality [11].

Male preponderance in this study is similar to the findings elsewhere [12–19]. This may be due to differences in certain risk factors such as smoking, which is much more prevalent among men in Nigeria compared with women.

Similar to other studies [20], in the present paper a high percentage of stroke in young adults were ischemic (59.1%). This finding is in support of the report by Onwuchekwa et al. in their study on stroke in young adult in Porthacourt [3]. It is also similar to the observation of stroke in the general population, where 71% of stroke patients had cerebral infarct in a study in southwestern Nigeria [21] and 63% at Maiduguri in northeastern Nigeria [22].

In the present paper, only 8.5% and 17% of the patients presented before 3 and 6 hours, respectively at the emergency unit. In view of the short time window (3–6 hours) for thrombolytic therapy, this finding has a significant implication for thrombolytic therapy in the management of infarctive stroke in our setting. Therefore, provision of facilities for thrombolytic therapy without adequate education on early presentation and infrastructural support for early presentation may not necessarily make much difference.

Regarding risk factors, as previously reported by other studies [23, 24], hypertension and cigarette smoking appeared to be the most common modifiable risk factors for both ischaemic and haemorhagic stroke. This observation, similar to previous studies [3, 4], corroborates the report from Framingham studies which showed that hypertension is a clear risk factor for stroke in both sexes and in all ages and races [25]. Other risk factors included smoking, hypercholesterolemia, cardiac diseases, and HIV in descending order of frequencies. However, the majority of the patients had multiple risk factors. It is worthy of note that in 8.5% of the patients recruited no risk factors were detected. This category of patients should, however, be viewed from the perspective of the limited resources for in-depth evaluation.

Similar to some previous studies [12–16], half of the patient had positive history of cigarette smoking. The Framingham Heart Study was among the first to assess the relation of smoking to type of stroke, number of cigarettes smoked, and the effect of stopping [26]. It concluded that smoking made a significant independent contribution to the risk of stroke generally and to brain infarction specifically. In a meta-analysis of 32 separate studies, Shinton and Beevers showed that cigarette smoking independently contributed to the incidence of stroke: the greatest risk was of subarachnoid haemorrhage, followed by cerebral infarction [27].

HIV is commonly a disease of the young adult who, among other things, engages in high-risk behaviors, such as unprotected heterosexual contact and intravenous drug abuse. Few of our patients that consented to HIV screening were reactive. Incidentally, the two patients with cocaine and amphetamine use also had HIV. HIV is increasingly becoming a common risk factor for stroke in Sub-Saharan Africa [28], where it has been shown to be associated with coagulation abnormalities, such as Protein S deficiency. Mochan et al. found the causes of stroke in HIV-positive stroke patients to be similar to those in HIV-negative stroke patients [29]. Nevertheless, whether HIV by itself actually causes or independently increases the risk of stroke has been a recurrent question even though it is known to cause an intracranial small vessel vasculopathy [29] as well as an extra-cranial large artery vasculitis [30, 31]. A study has fairly convincingly found HIV to be an independent risk for stroke [28]. Potential etiologies for vascular disease among HIV-infected patients include an underlying viral myocarditis, congestive cardiomyopathy, infective endocarditis, atheroma, and thromboembolism from arterial plaques.

Sickle cell disease and connective tissue diseases should also be given consideration when dealing with stroke in young adult in developing countries as shown in the present study to have accounted for (2.8%) of cases of stroke recruited.

Mortality and case fatality in the present study are comparable to what was reported in the other geopolitical zones of Nigeria [3–5, 22].

 Aspiration pneumonitis being a common finding associated with death is worthy of note; this is in conformity with a study by Hassan et al. reported that 23% of patients with stroke developed stroke-associated pneumonia, of which 34% died during hospital stay [32, 33]. Strict adherence to swallow test on all stroke patients, proper adequate nursing care, and ultimately establishment of stroke unit are measures that could dramatically reduce aspiration-related mortality in our setting.

Regarding disability in survivors, comparing the degree of disability at discharge to disability at 6-month followup, some improvement was recorded. This can be largely ascribed to intensive and qualitative physiotherapy and secondary prevention of stroke.

Nevertheless, it suffices to state that this study suffers from some limitations, being a hospital-based study limits its external validity. Moreover, error of misclassification cannot be completely ruled out as neuroimaging was done in all the patients. However, the findings provide some clues as to the pattern, clinical characteristics, and case fatality of stroke in young adult in Kano. Besides, it generated a background data and impetus for a larger community-based study on stroke in the young in Kano.

## 5. Conclusion

Our data suggests that stroke in young adults is not as uncommon as previously suggested, and that hypertension, smoking, hypercholesterolemia, cardiac diseases and HIV are the most common risk factors. Infarctive stroke was commoner than haemorhagic stroke, and the overall case fatality was high (19.7%) in the first seventy-two hours. The study also showed increasing frequency of HIV as a risk factor for stroke among young adults in the study area.

## Figures and Tables

**Table 1:** Distribution of stroke type and sex by age group.

Age group	Infarctive		Haemorhagic		Total
	Male	Female	Male	Female	
10–20	3	—	—	—	3
21–30	16	2	8	1	27
31–40	10	11	15	5	41

Total	29	13	23	6	71

**Table 2:** Delay before presentation in hours.

Delay before presentation (Hours)	Frequency	Percent
<3	6	8.5
3–6	6	8.5
7–24	23	32.4
25–48	33	46.2
>48	3	4.2

Total	71	100

**Table 3:** Traditional risk factors*.

Risk factors	Frequency	Percentage
Hypertension	53	74.7
Smoking	36	50.7
Hypercholesterolemia	7	9.9
Cardiac diseases**	6	8.5
HIV	6	8.5
Previous stroke and or transient ischaemic attack	4	5.6
Diabetes	3	4.2
Alcohol	3	4.2
Migraine	3	4.2
Drug of addiction (cocaine, amphetamine)	2	2.8
Sickle cell disease	2	2.8
Connective tissue disease	1	1.4
Unidentified	6	8.5

*Some patients had multiple risk factors.

**Nonhypertensive heart diseases (rheumatic and nonrheumatic valvular heart diseases).

**Table 4:** Status of patients on modified ranking scale on discharge and at 6-month followup.

Ranking disability scale	Frequency at discharge from hospital	Frequency at 6-month followup*
0 (no symptoms)	—	2
1 (no significant disability)	1	4
2 (slight disability)	11	11
3 (moderate disability)	16	10
4 (moderately severe disability)	11	6
5 (severe disability)	9	4

Total	48	37

*11 patients were lost to followup.
